# Causal roles and clinical utility of cardiovascular proteins in colorectal cancer risk: a multi-modal study integrating mendelian randomization, expression profiling, and survival analysis

**DOI:** 10.1186/s12920-024-01909-4

**Published:** 2024-05-22

**Authors:** Chenlei Tan, Yanhua Li, Kexin Wang, Ying Lin, Yu Chen, Xuebao Zheng

**Affiliations:** 1 The Second School of Clinical Medicine of Zhejiang Chinese Medical University, No. 548 Binwen Road, Binjiang District, Hangzhou, 310053 Zhejiang P. R. China; 2 General Practice Department at the Second Hospital of Zhejiang Chinese Medical University, No. 318 Chaowang Road, Hangzhou, 310005 Zhejiang P. R. China

**Keywords:** Cardiovascular, Plasma proteins, Colorectal cancer risk, Mendelian randomization, Gene expression profiling, Survival analysis, Therapeutic targets

## Abstract

**Purpose:**

This comprehensive investigation delved into the intricate causal interplay existing between cardiovascular-related plasma proteins and the susceptibility to colorectal cancer, leveraging the robust framework of Mendelian randomization, and employed expression profiling and survival analysis to unravel the latent clinical worth embedded within pertinent gene expressions.

**Methods:**

Protein quantitative trait loci (pQTLs) of 85 cardiovascular proteins were employed as instrumental variables to investigate the causal relationship between proteins and CRC risk using a Mendelian randomization approach. Causal inferences were graded as strong, intermediate or weak based on statistical checks. Drug-target MR examined VEGF receptors for their potential as therapeutic targets for colorectal cancer. Differential expression analysis, diagnostic ROC curves, and survival analyses were performed for identified proteins using RNA-seq data from The Cancer Genome Atlas (TCGA) colorectal cancer cohort.

**Results:**

Using cis-pQTLs, LOX-1, VEGF-A and OPG were associated with increased CRC risk (strong evidence), while PTX3, TNF-R2 and MMP-7 were protective (strong evidence). Pan-pQTL analysis found MMP-10 increased risk (intermediate evidence) and ADM increased risk (weak evidence). Drug-target MR found VEGF R1 may be promising therapeutic targets. Differential expression analysis revealed seven genes encoding the identified proteins were dysregulated in tumors. ROC analysis showed five gene expression had high diagnostic accuracy. KM analysis showed four genes had prognostic value.

**Conclusions:**

This large-scale MR study implicates several cardiovascular proteins in CRC susceptibility and progression. Findings highlight roles for VEGF signaling and extracellular matrix regulation. Results nominate specific proteins as potential diagnostic biomarkers or therapeutic targets warranting further investigation.

**Supplementary Information:**

The online version contains supplementary material available at 10.1186/s12920-024-01909-4.

## Introduction

Colorectal cancer (CRC), is a major global health concern, ranking third in global incidence and second in mortality among all types of cancer [10, 27]; [33]. CRC is a neoplastic ailment characterized by the aberrant cellular proliferation occurring within the epithelial tissues of the colon and rectum [4] Its emergence is frequently attributed to a plethora of genetic mutations and impairments in the intricate machinery responsible for DNA repair mechanisms [1] The pathogenesis of CRC typically follows a multistep paradigm, encompassing the sequential advancement from a state of normalcy within the epithelial tissues, to the formation of precancerous lesions, culminating in the invasive carcinoma stage [24]. Clinically, CRC often presents with symptoms such as blood in the stool, changes in bowel habits, abdominal pain, and other symptoms like fatigue, decreased appetite, fever and nausea [12]. Numerous etiological determinants underlie the pathogenesis of colorectal carcinoma (CRC), encompassing heritable predispositions, individual and familial anamnesis, coexisting medical conditions, as well as modifiable lifestyle attributes including dietary patterns, levels of physical exertion, tobacco usage, and alcohol indulgence [9, 17, 21]. Despite advances in treatment, late diagnosis and the limited effectiveness of chemotherapy remain significant challenges [6, 11]. Thus, more research is needed to develop less aggressive and more effective treatment strategies, and to fully understand the underlying genetic and molecular mechanisms of CRC.

Cardiovascular disease (CVD) remains a leading cause of morbidity and mortality worldwide [7]. Cardiovascular related proteins have been extensively studied in relation to the occurrence and development of CVD, and have been shown to be useful biomarkers for identifying individuals at risk of developing the disease [8]. In recent years, there has been increasing interest in the potential link between cardiovascular related proteins and the development of other diseases, including cancer [13, 19]. Several studies have reported that certain cardiovascular related proteins are also involved in the occurrence and development of CRC [18, 26; 2]. These findings suggest that the biomarkers related to CVD may be associated with the risk of CRC, and that investigating the potential biological pathways underlying these associations could provide valuable insights into the mechanisms by which these two diseases are linked. If our hypothesis is confirmed, our findings could have important implications for the development of new strategies for the prevention and treatment of both diseases. In particular, the identification of novel biomarkers that could be used to identify individuals at high risk of developing both CVD and CRC could have significant clinical and public health implications. Ultimately, our study highlights the importance of investigating the relationships between different diseases and the potential shared mechanisms that underlie their pathogenesis.

In this study, we aim to investigate the relationship between cardiovascular related proteins and the risk of CRC. Specifically, we utilized a Mendelian randomization approach to investigate the causal relationship between 85 cardiovascular proteins and CRC. Specifically, we selected the corresponding cis- and trans-pQTLs of the proteins as instrumental variables (IVs) and used CRC as the outcome. After testing for horizontal pleiotropy and heterogeneity, we obtained positive results. Furthermore, drug-target MR was employed for therapeutic targets of CRC. In addition, we also investigated the differential expression of the target proteins’ mRNA between CRC and healthy controls and explored their clinical significance in diagnosis and prognosis. Our results indicate a causal relationship between certain cardiovascular proteins and CRC, suggesting that these proteins may play a role in the development of the disease. Furthermore, our analysis of mRNA expression levels provides additional evidence supporting this link. Additionally, these findings may inform the development of new therapeutic targets for the treatment of CRC.

## Results

In this study, a total of 545 pQTLs (including 174 cis-pQTLs and 371 trans-pQTLs) were considered for 85 cardiovascular proteins. Two sets of instrumental variables, cis- and pan-pQTLs, were used for Mendelian Randomization analysis with CRC as the outcome. Using cis-pQTLs as instrumental variables, LOX-1 (OR = 1.82 (1.18–2.80), *P* = 6.8e-3), VEGF-A (OR = 1.10 (1.02–1.19), *P* = 0.02), and OPG (OR = 1.22 (1.02–1.47), *P* = 0.03) were found to increase the risk of CRC, while PTX3 (OR = 0.59 (0.40–0.88), *P* = 0.01), TNF-R2 (OR = 0.68 (0.49–0.93), *P* = 0.02), and MMP-7 (OR = 0.88 (0.78-1.00), *P* = 0.04) were found to be protective. When pan-pQTLs were used as instrumental variables, ADM (OR = 1.38 (1.08–1.77), *P* = 9.1e-2) and MMP-10 (OR = 1.19 (1.00-1.43), *P* = 0.04) were found to increase the risk of CRC.

**Fig. 1:**
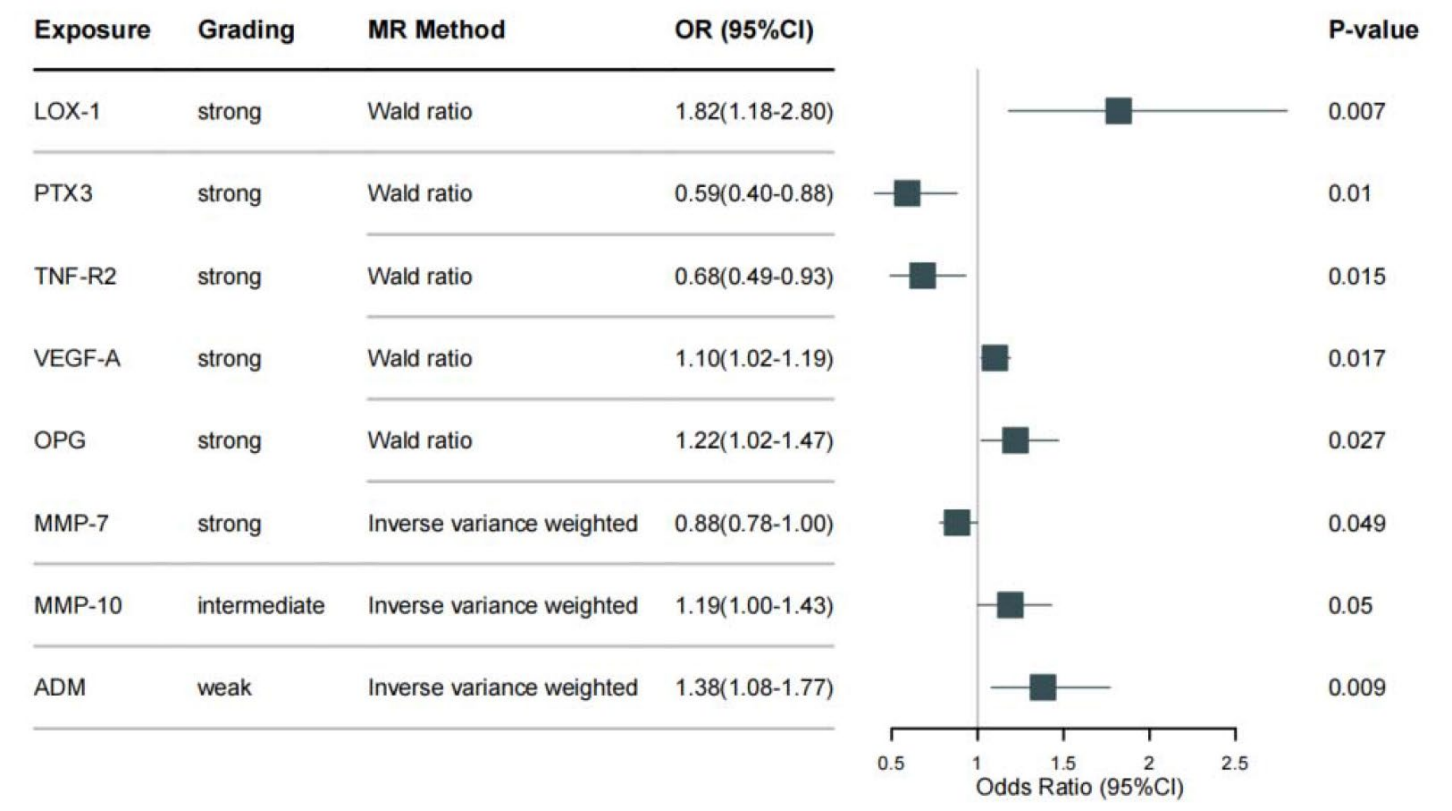
Associations of proteins with CRC risk: findings from mendelian randomization analysis

In this study, we conducted a sensitivity analysis to evaluate the robustness of our Mendelian Randomization results obtained using cis- and trans-pQTLs as instrumental variables (IVs). To this end, we performed a search for results using cis-pQTLs as IVs, with a focus on identifying SNPs that were also cis- or trans-pQTLs, splicing QTLs (sQTLs), or expression QTLs (eQTLs) of other disease risk-influencing genes. Additionally, we searched the PhenoScanner database for significant SNP-trait associations (*p* < 5 × 10^− 8^) documented in over 5000 GWASs at the time of the study. Our search did not reveal any evidence of horizontal pleiotropy caused by the identified SNPs. As a result, we classified the six resulting associations as ‘strong’. For the results obtained using pan-pQTLs as IVs, we performed tests for both horizontal pleiotropy and heterogeneity. Our analysis did not identify any evidence of horizontal pleiotropy or heterogeneity for MMP-10. Similarly, no evidence of heterogeneity was found for ADM. However, due to the limited number of IVs, we were unable to test for pleiotropy in the case of ADM. Therefore, we classified the association for MMP-10 as ‘intermediate’, while that for ADM was classified as ‘weak’. Details of all SNPs employed as IVs in this section and their F values were summarized in Table S1. A summary of all of our results can be found in Fig. 1, with additional details regarding heterogeneity, pleiotropy, and leave-one-out results provided in the Figure S1 and Table S2.

VEGF has been identified as a crucial contributor to the pathogenesis of tumors, rendering it a significant target for cancer treatment [34]. VEGFR, the receptor for VEGF, binds to VEGF and propagates signals that regulate cellular proliferation, survival, and motility [22]. A plethora of drugs have been designed and utilized in the treatment of cancer, with significant research dedicated to VEGF inhibitors and VEGFR inhibitors [5, 16, 23, 25]. Given our prior findings that VEGF-A may influence CRC, we conducted a drug-target mendelian randomization study to investigate the potential of VEGFR inhibitors as therapeutic agents for this cancer. When utilizing cis-IVs for Mendelian randomization analysis, the results indicated no significant associations between VEGFR2, VEGFR3, and colorectal cancer (CRC). The odds ratios (OR) with their respective 95% confidence intervals (CI) were as follows: VEGFR2 OR (95% CI) = 1.03 (0.95–1.13), *p* = 0.44, using the Wald ratio test; VEGFR3 OR (95% CI) = 1.01 (0.98–1.18), *p* = 0.17, using the Wald ratio method. Additionally, when utilizing pan-IVs, which include both cis- and trans-pQTLs, for MR analysis, the results similarly showed no significant associations between VEGFR2, VEGFR3, and CRC. The OR with their respective 95% CI were as follows: VEGFR2 OR (95% CI) = 1.02 (0.97–1.08), *p* = 0.44; VEGFR3 OR (95% CI) = 1.03 (0.99–1.08), *p* = 0.17, using inverse variance weighted method. Due to the absence of pQTLs for VEGFR1 (also known as VEGF sR1), we proceeded to use the cis-eQTLs of the FLT1 gene, which encodes VEGFR1, as instrumental variables for MR analysis. In contrast, by utilizing the lead cis-eQTL as an instrumental variable to study VEGF R1, we observed a reduction in the risk of CRC (OR (95% CI) = 0.81 (0.71–0.93), *P* = 2.1E-3). Furthermore, the lead cis-eQTL rs56728557 did not exhibit any significant associations with any phenotype or disease in PhenoScanner, thereby enhancing the reliability of our findings. Based on these results, we posited that VEGF sR1 could serve as a promising target for the treatment of CRC, and drugs aimed at targeting it may have clinical utility. Details of all SNPs employed as IVs in this section and their F values were summarized in Table S3.

**Fig. 2:**
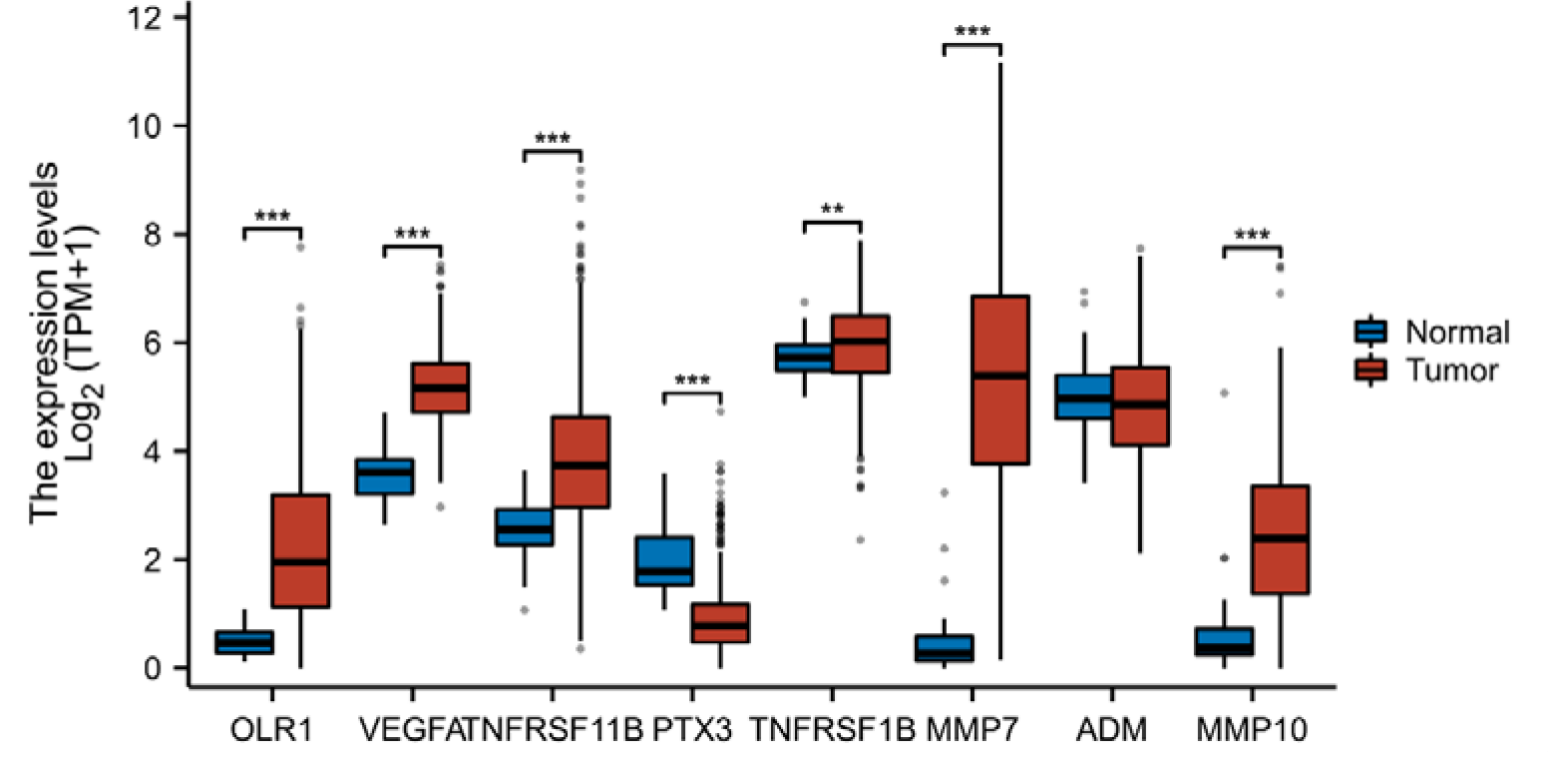
Differential expression of 8 target protein genes between cancer and healthy controls

**Fig. 3:**
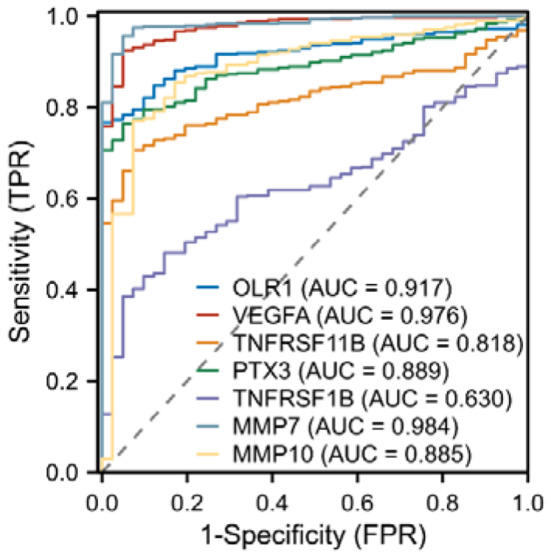
ROC curves of 6 target protein genes based on clinical information

**Fig. 4:**
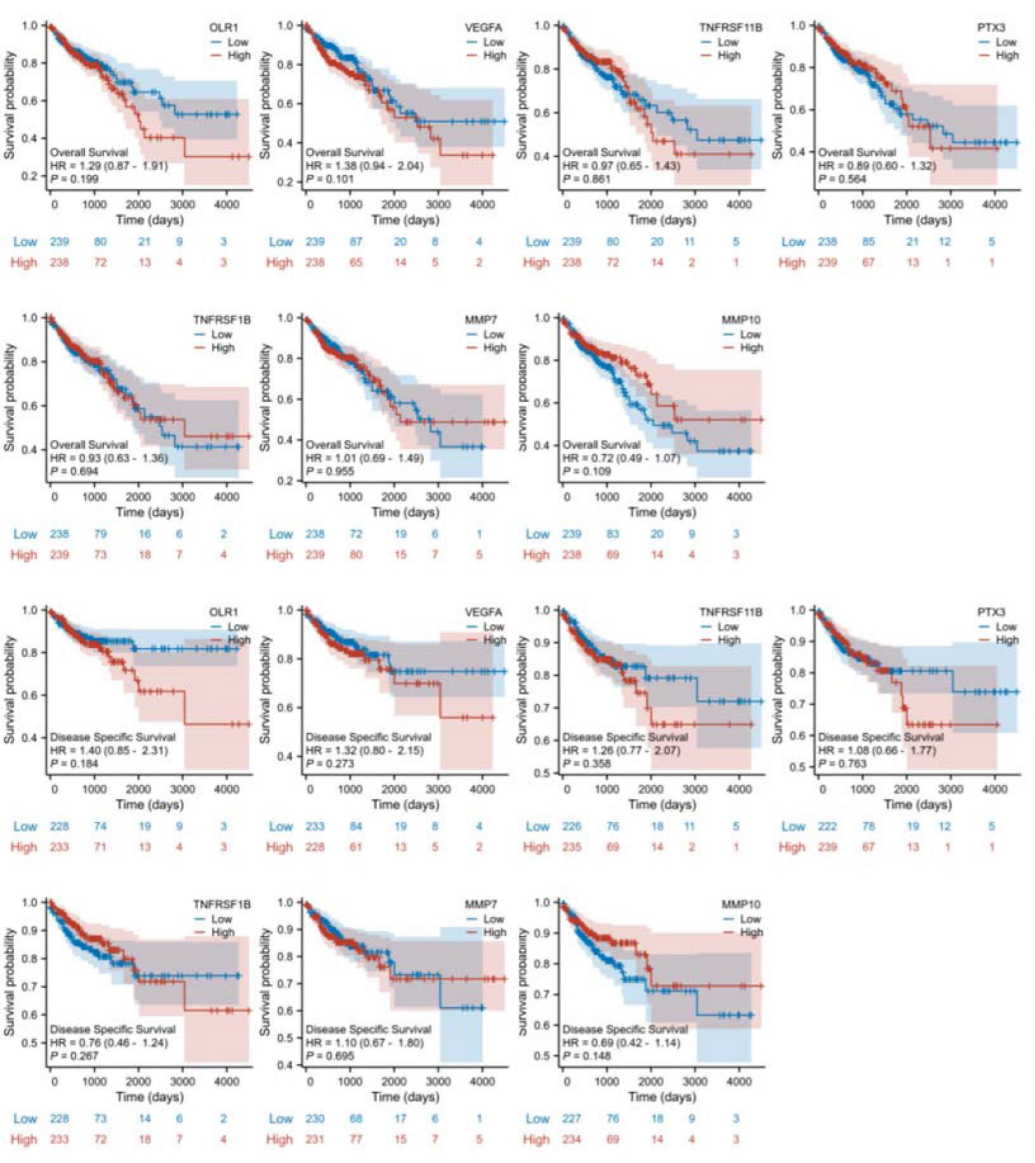
KM curves of seven target protein genes based on clinical information

Subsequently, we investigated the differential expression of these eight genes between tumor and normal tissues. The genes included LOX-1 (OLR1 [ENSG00000173391.9]), VEGF-A (VEGFA [ENSG00000112715.25]), OPG (TNFRSF11B [ENSG00000164761.9]), PTX3 (PTX3 [ENSG00000163661.4]), TNF-R2 (TNFRSF1B [ENSG00000028137.19]), MMP-7 (MMP7 [ENSG00000137673.9]), ADM (ADM [ENSG00000148926.10]), and MMP-10 (MMP10 [ENSG00000166670.10]). The results are presented in Fig. 2 and Table S4, which revealed that OLR1, VEGFA, TNFRSF11B, TNFRSF1B, MMP7, and MMP10 were significantly upregulated in tumor tissues, while PTX3 was significantly downregulated in tumor tissues. To verify the diagnostic value of these seven dysregulated genes, we plotted diagnostic ROC curves, as shown in Fig. 3 and Table S5. OLR1, VEGFA, PTX3, MMP7, and MMP10 all demonstrated good diagnostic performance in predicting outcomes, with high accuracy AUCs of 0.917 (CI = 0.891–0.943), 0.976 (CI = 0.960–0.992), 0.889 (CI = 0.858–0.920), 0.984 (CI = 0.972–0.995), and 0.885 (CI = 0.831–0.939), respectively. To evaluate the prognostic effect of the expression of these six genes, we plotted KM curves of OS and DSS, as shown in Fig. 4. Among them, OLR1 and MMP10 in OS, OLR1, TNFRSF11B, PTX3 and MMP10 in DSS show some prognosis value, due to their involvement in the occurrence and development of CRC.

## Discussion

This study provides novel evidence for causal associations between cardiovascular-related plasma proteins and CRC risk using a Mendelian randomization approach. Specifically, our results suggest LOX-1, VEGF-A, OPG, ADM, and MMP-10 may increase CRC risk, while PTX3, TNF-R2, and MMP-7 may be protective against CRC. We then performed drug-target Mendelian randomization using cis-pQTLs and cis-eQTLs as instrumental variables for the VEGF receptors. This analysis suggested VEGF R1 may be promising therapeutic targets for CRC treatment. We further validated the differential expression of these proteins between CRC and normal colon tissues using RNA-sequencing data, revealing significant dysregulation in CRC. Finally, we demonstrated the diagnostic and prognostic potential of the identified proteins.

Notably, many of the identified proteins play established roles in pathways relevant to cancer development, providing biological plausibility for the detected associations. For instance, LOX-1, a receptor for oxidized LDL, has been shown to promote tumor proliferation, migration, and invasion in several cancer types including CRC [19]. High levels of serum LOX-1 were associated with significantly poorer overall survival, and LOX-1 was identified as an independent prognostic factor in liquid biopsy [20]. VEGF-A, one of the most critical and specific factors that stimulate both physiological and pathological angiogenesis, is a key regulator of angiogenesis, a process critical for tumor growth [15]. A study investigated the correlation between serum levels of EphA2 and VEGF-A and the pathogenesis of CRC, as well as the potential value of these molecules in the diagnosis of CRC, suggesting that the serum level of VEGF-A can be used as a potential serological marker for the diagnosis of CRC [31]. OPG, also known as tumor necrosis factor receptor superfamily member 11B (TNFRSF11B), is characterized by its ability to bind to receptor activator of nuclear factor kappa B ligand (RANKL) and plays a critical role in bone remodeling [32]. The mRNA expression of OPG in cancer tissues was significantly higher in patients with distant metastases than those without metastases. Overexpression of OPG protein was associated with significantly worse overall survival and relapse-free survival, and was identified as an independent risk factor for CRC recurrence [29]. PTX3, a crucial component of innate immunity, serves as an extrinsic oncosuppressor by regulating Complement-mediated, macrophage-driven inflammation, influencing resistance against microbes, inflammation modulation, and tumor susceptibility [3].

A key strength of our study was the use of a MR approach utilizing cis- and trans- pQTLs as genetic instruments to minimize biases from confounding and reverse causation. We further conducted sensitivity analyses to assess for pleiotropy and heterogeneity. The differential expression results provide orthogonal evidence linking these proteins to CRC transcriptomic dysregulation. Additional strengths include the large sample sizes and extensive pQTL data from SCALLOP. However, some limitations should be noted. We could not definitively rule out pleiotropy for proteins with trans-pQTL instruments. The TCGA expression data may not fully reflect peripheral protein levels. Further experimental studies are needed to dissect the functional roles of identified proteins in CRC pathogenesis. Our study was limited to individuals of European ancestry, and replication is needed in other populations. CRC is a heterogeneous disease, and stratification by molecular subtypes may reveal additional insights.

In summary, our integrative genomics study reveals potentially causal associations between cardiovascular-related plasma proteins and CRC risk. Drug-target MR found VEGF R1 as a potential therapeutic target for CRC. The identified proteins may serve as promising diagnostic, prognostic, and therapeutic targets worthy of further investigation and validation. Our work highlights the interconnected nature of the plasma proteome and comorbid disease states. Additional phenome-wide MR studies in large biobanks may uncover more causal protein-disease associations spanning diverse physiologic systems.

## Conclusion

In conclusion, our study provides novel evidence for causal associations between cardiovascular-related plasma proteins and risk of colorectal cancer using an integrative genomics approach. Specifically, we identified LOX-1, VEGF-A, OPG, ADM, and MMP-10 as potential causal risk factors for CRC, while PTX3, TNF-R2, and MMP-7 may be protective, VEGF R1 as a potential drug target based on results from Mendelian randomization analyses. We further demonstrated significant dysregulation of these proteins in CRC tissues compared to normal colon samples. Several of the proteins also showed promise as diagnostic and prognostic biomarkers. The implicated proteins are involved in pathways highly relevant to cancer, including angiogenesis, inflammation, cell proliferation, invasion and metastasis. Our findings suggest these proteins may contribute to CRC development through multiple independent biological mechanisms. Additional studies are warranted to replicate these results in diverse populations and elucidate the precise functional roles of these cardiovascular-related proteins in colorectal tumorigenesis. Experimental validation of the identified proteins as therapeutic targets may ultimately lead to new strategies for preventing and treating CRC. More broadly, our study highlights the power of utilizing proteomic quantitative trait loci in Mendelian randomization frameworks to elucidate causal associations between plasma proteins and disease outcomes. Further mining of the plasma proteome through such unbiased approaches may uncover novel inter-relationships between circulating proteins and disparate pathophysiologic states. This could lead to new diagnostics, drug targets, and insights into molecular connections spanning the human phenome.

## Method

### Data resource

(1) All cis- and trans-pQTLs of 85 cardiovascular proteins were obtained from a study by Folkersen et al. which maps and replicates pQTLs for 90 cardiovascular proteins in over 30,000 individuals, resulting in 545 pQTLs (Folkersen, et al. 2020). The per-allele effects of pQTLs could all be transformed to reflect the predicted per-standard-deviation (SD) change in the protein level in the population. These results are from the SCALLOP consortium, a collaborative framework for pQTL mapping and biomarker analysis of proteins on the Olink platform (https://www.olink.com/scallop/). (2) Summary GWAS statistics for outcomes were obtained from data published by the FinnGen study (https://www.finngen.fi/, phenocode: R9_C3_COLORECTAL_EXALLC) [14]. This GWAS included 293,646 European adults, 6,509 cases, and 287,137 healthy controls. A total of 20,175,454 SNPs (single nucleotide polymorphisms) were included in the study. (3) cis-pQTLs of vascular endothelial growth factor receptor 2 (VEGF sR2) and vascular endothelial growth factor receptor 3 (VEGF sR3) were obtained from the genomic atlas of the human plasma proteome [28]. Cis-eQTLs of FLT1 gene encoding vascular eSndothelial growth factor receptor 1 (VEGF R1) were obtained from eQTLGen consortium (https://eqtlgen.org/) [30]. (4) The RNA-seq transcriptome data and pertinent clinical details encompassing gender, age, subtype, IDH status, and survival information of CRC were procured from The Cancer Genome Atlas (TCGA)-COAD database (https://portal.gdc.cancer.gov/). All data generated or analyzed during this study are publicly available, so our study doesn’t need ethics declaration or clinical trial number.

**Fig. 5:**
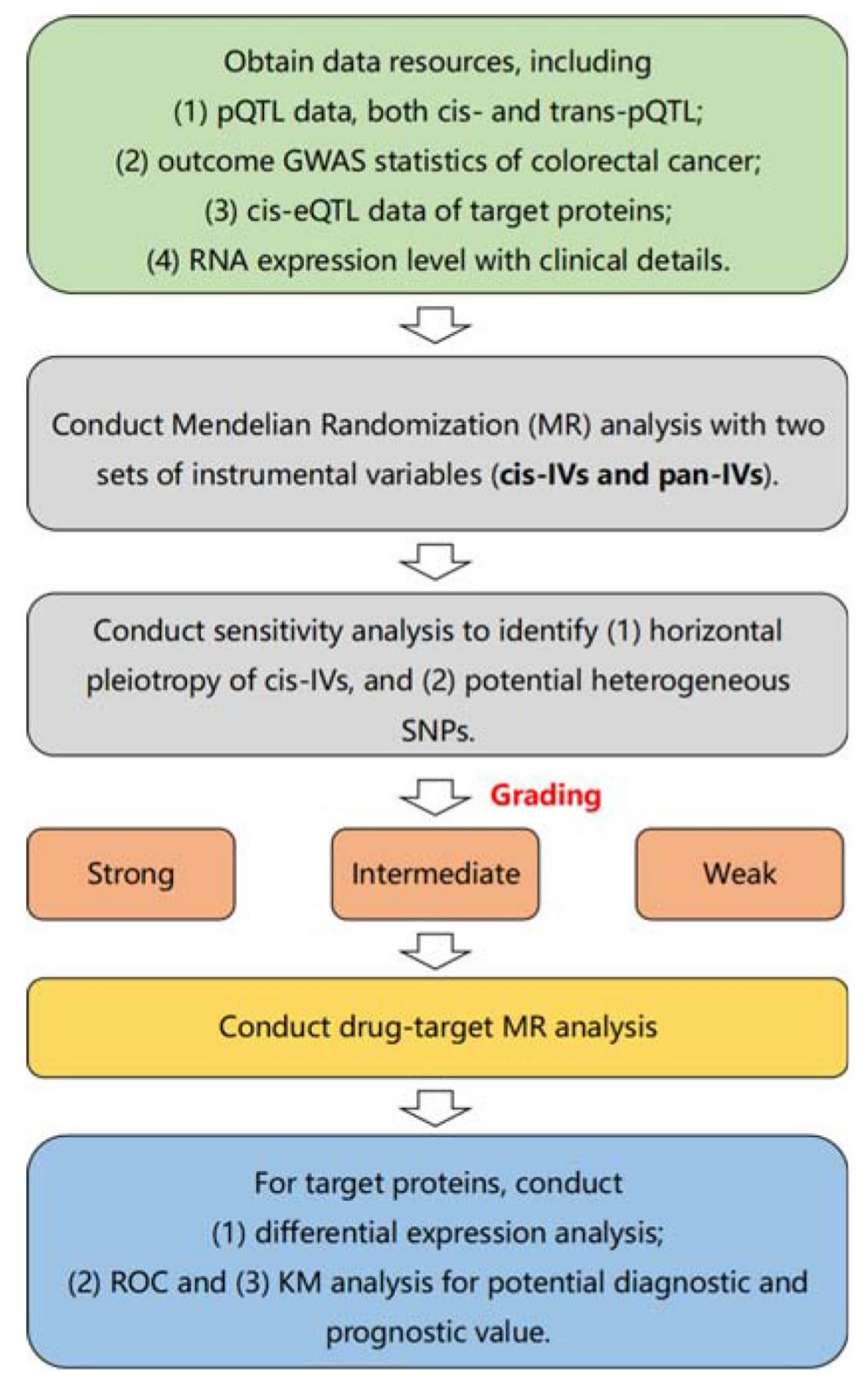
The flow chart of the analysis procedure in this study

### Mendelian randomization

Mendelian randomization is a powerful approach for examining causality in observational studies, as it employs genetic variants that are randomly assigned at conception and thus not influenced by environmental or lifestyle factors, thereby avoiding confounding. In our investigation, we employed pQTLs as instrumental variables (IVs), which have been shown to be dependable and robust instruments for Mendelian randomization analyses of protein biomarkers. However, the accuracy of causal inference in MR depends on several fundamental assumptions. the first critical assumption is that there is a significant association between the genetic variation used as an IV and the exposure of interest. This assumption is crucial, as it ensures that the genetic variation is a reliable surrogate for the exposure, allowing for the estimation of a causal effect. Violation of this assumption can lead to biased estimates of the causal effect. The second fundamental assumption of MR is that the genetic variation used as an IV is not linked to any confounding factors that could confound the relationship between exposure and outcome. The third assumption of MR is that the genetic variant used as an IV has no other effects on the outcome apart from its impact on the exposure. This assumption posits that the genetic variant used as an IV only affects the outcome through its effect on the exposure and not through any other pathways.

To investigate the causal effects of proteins on outcomes, we conducted two-sample MR analyses in our study, which involved creating two sets of instrumental variables (IVs) for each of the 85 proteins with variants reaching multiple-testing-corrected significance in the original discovery GWAS. The first set of IVs, known as cis-IVs, consisted of one or more independent variants (with LD r^2^ = 0.001) located within ± 1 Mb of the transcript boundaries of the gene encoding the protein. The second set of IVs, known as pan-IVs, included all independent variants (with LD r^2^ = 0) associated with the protein, combining both cis- and trans-pQTLs. For each individual SNP-protein and SNP-outcome association, we generated an IV Wald ratio estimate. When the instrument contained more than one SNP, we generated summary IV estimates by combining individual-SNP Wald estimates using the inverse variance-weighted (IVW) method. In the absence of horizontal pleiotropy, the IVW results would be unbiased. We used the TwoSampleMR (version 0.5.6) R package for our MR analysis in this study.

In our study, we selected proteins associated with CRC for further sensitivity analysis. Firstly, we investigated the possibility of horizontal pleiotropy, which occurs when the instrumental variable estimation affects the outcome through factors other than exposure, resulting in a violation of the independence and exclusivity assumptions. To identify horizontal pleiotropic effects, we examined whether the selected cis-pQTL of a target protein was mostly a cis- or trans-pQTL, splicing QTL(sQTL), or expression QTL(eQTL) of another disease risk influencing gene. We also searched for significant SNP-trait associations (*p* < 5 × 10^− 8^) documented in the PhenoScanner database, which included over 5000 GWASs at the time of the study. Secondly, we employed MR Egger regression for proteins with at least 3 genetic instruments, where a significant MR Egger expression intercept (*p* < 0.05) would also indicate the presence of horizontal pleiotropy. Thirdly, we considered the potential heterogeneity of instrumental variables (IVs) from different analysis platforms, experiments, populations, etc., which could affect the results of Mendelian randomization’s randomized analysis. To quantify the heterogeneity of IVs, we used Cochran’s IVW Q statistics. Finally, to enhance the robustness of the analysis, we employed a leave-one-out validation approach, where each instrumental SNP was removed in turn, to identify potential heterogeneous SNPs.

### MR result grading

In our study, we have established a classification system for assessing causality evidence into three distinct categories. (1) The “strong” category is defined by both the presence of a cis-IV estimate that achieves statistical significance (*P* < 0.05) and no discernible heterogeneity or pleiotropic effects, suggesting a robust and direct causal relationship. The “intermediate” category is identified through a pan-IV estimate, which combines both cis- and trans-pQTLs of proteins, again with a significance level of *P* < 0.05 and no discernible heterogeneity or pleiotropic effects, indicating a probable but potentially less direct causal inference. Because pan-IV estimate also employed trans-pQTLs as IVs, which map to genes that do not directly code for the targeted proteins or to intergenic regions. Finally, the “weak” category encompasses results where either a cis-IV or pan-IV estimate reaches statistical significance (*P* < 0.05), but there are insufficient IVs to adequately test for heterogeneity or pleiotropy. Finally, results indicating heterogeneity or pleiotropy, even if statistically significant (*P* < 0.05), are excluded from further analysis, as these may confound the causal interpretation.

### Drug-target mendelian randomization

Cis-pQTLs were used as IVs for drug-target mendelian randomization of VEGF sR2 and VEGF sR3. Since no suitable cis-pQTLs was found for VEGF R1, we collected the cis-eQTLs of FLT1 ([ENSG00000102755]) gene encoding VEGF R1, consistent of one or more independent variants (with LD r^2^ = 0.001) located within ± 1 Mb of the transcript boundaries of the gene encoding the protein, and screened out the lead cis-eQTL, which is defined as the cis-eQTL showing the strongest association with gene expression, as the IV for mendelian randomization. Other details of the MR method were consistent with the above.

### Differential expression of the target protein genes and their potential clinical significance

Our study conducted a differential expression analysis of the target protein gene expression between cancer and healthy controls using the RNAseq data of the STAR process from the TCGA-COAD project, which was downloaded and organized from https://portal.gdc.cancer.gov. The TPM format data was extracted and processed using log_2_ (value + 1). The statistical method employed was the Wilcoxon rank sum test, and the data was visualized using the ggplot2 package (version 3.3.6). To investigate the diagnostic significance and predictive accuracy of target protein expression, we performed ROC analysis using the pROC package (version 1.18.0), and the results were visualized using the ggplot2 package (version 3.3.6). To compare the prognosis between groups with different expression levels, we utilized cox regression for statistics and plotted the KM survival curve. The proportional risk hypothesis was tested using the survival package (version 3.3.1) and fitted survival regression. The results were visualized using the survivor package and the ggplot2 (version 3.3.6) package. The data filtering strategy involved removing normal samples and those without clinical information. The prognosis types considered were OS (overall survival) and DSS (disease-specific survival), and the median grouping method was employed.

The flow chart of this analysis for our study is shown in Fig. 5.

### Electronic supplementary material

Below is the link to the electronic supplementary material.

Supplementary Material 1

## Data Availability

The database is publicly available. All data generated or analyzed during this study are included in this published article and its supplementary information files. All the pQTLs for 85 cardiovascular proteins used during this study are included in this published article (*Nat Metab 2*, **2020**, 1135–1148) The GWAS summary data for CRC during the current study are available in the website (https://www.finngen.fi/en/access_results). The data for differential expression and clinical significance analysis during the current study are available in the website (https://portal.gdc.cancer.gov/). All the analysis are completed in the R (4.1.3).
