# Supplementary material for: Causal roles and clinical utility of cardiovascular proteins in colorectal cancer risk: a multi-modal study integrating mendelian randomization, expression profiling, and survival analysis
Source: BMC Med Genomics. 2024 May 22;17:138. doi: 10.1186/s12920-024-01909-4 (PMC11110250; doi:10.1186/s12920-024-01909-4)
Supplement: Supplementary file 1 — Supplementary Material 1 [file 12920_2024_1909_MOESM1_ESM.docx]

**Supplementary Information (SI) for**

**Causal Roles and Clinical Utility of Cardiovascular Proteins in Colorectal Cancer Risk: A Multi-modal Study Integrating Mendelian Randomization, Expression Profiling, and Survival Analysis**

Chenlei Tan,^1#^ Yanhua Li,^2#^ Kexin Wang,^1#^ Ying Lin,^1^ Yu Chen,^1^ Xuebao Zheng,^1^ Yanhua Li^2*^

^1^ The Second School of Clinical Medicine of Zhejiang Chinese Medical University, No. 548 Binwen Road, Binjiang District, Hangzhou, 310053, Zhejiang, P. R. China.

^2^ General Practice Department at the Second Hospital of Zhejiang Chinese Medical University, No. 318 Chaowang Road, Hangzhou, 310005, Zhejiang, P. R. China.

*** Correspondence Author:**Yanhua Li (Liyanhua0330@163.com)

**# C. Tan, Y. Li and K. Wang contributed equally to this manuscript.**

**Table S1** All IVs of cardiovascular proteins for mendelian randomization

|  |  |  |  | exposure | | | outcome | | |  |  |
| --- | --- | --- | --- | --- | --- | --- | --- | --- | --- | --- | --- |
| protein | SNP | effect allele | other allele | beta | se | p | beta | se | p | F value | type of IVs |
| ADM | rs2218793 | A | C | -0.09 | 0.01 | 1.10E-13 | 0.02 | 0.02 | 0.23 | 53.78 | cis |
| ADM | rs4494298 | A | G | -0.16 | 0.01 | 1.20E-42 | -0.05 | 0.02 | 0.02 | 211.57 | cis |
| AGRP | rs138539707 | T | C | 0.23 | 0.04 | 5.80E-10 | -0.05 | 0.07 | 0.41 | 38.64 | cis |
| CASP-8 | rs35550815 | A | G | -0.19 | 0.02 | 6.00E-26 | -0.01 | 0.03 | 0.82 | 111.42 | cis |
| CCL20 | rs4973334 | T | C | -0.09 | 0.01 | 9.00E-15 | 0.03 | 0.02 | 0.12 | 62.55 | cis |
| CCL3 | rs1634517 | A | C | 0.40 | 0.01 | 1.00E-200 | -0.02 | 0.02 | 0.39 | 1111.11 | cis |
| CCL3 | rs9911791 | A | G | 0.13 | 0.01 | 3.10E-20 | 0.03 | 0.02 | 0.16 | 86.22 | cis |
| CCL4 | rs79943102 | T | C | 0.22 | 0.02 | 2.00E-24 | 0.01 | 0.03 | 0.71 | 100.00 | cis |
| CCL4 | rs8064426 | A | G | -0.44 | 0.02 | 3.20E-172 | -0.02 | 0.02 | 0.37 | 756.25 | cis |
| CHI3L1 | rs4950931 | A | G | 0.67 | 0.01 | 1.00E-200 | 0.02 | 0.02 | 0.40 | 4489.00 | cis |
| CSF-1 | rs11579145 | A | G | -0.18 | 0.01 | 7.30E-56 | -0.01 | 0.02 | 0.63 | 267.77 | cis |
| CSTB | rs35285321 | A | G | -0.53 | 0.01 | 1.00E-200 | 0.02 | 0.02 | 0.33 | 2809.00 | cis |
| CTSD | rs55861089 | A | G | 0.43 | 0.02 | 5.50E-166 | -0.02 | 0.04 | 0.57 | 722.27 | cis |
| CTSL1 | rs2274611 | T | C | -0.12 | 0.01 | 4.80E-30 | 0.02 | 0.02 | 0.20 | 144.00 | cis |
| CX3CL1 | rs683544 | T | C | -0.20 | 0.01 | 2.80E-73 | 0.02 | 0.02 | 0.32 | 330.58 | cis |
| CXCL16 | rs35186877 | A | G | -0.17 | 0.01 | 6.40E-42 | -0.01 | 0.02 | 0.74 | 200.69 | cis |
| CXCL16 | rs4790231 | A | G | 0.10 | 0.01 | 1.10E-21 | 0.00 | 0.02 | 0.84 | 92.08 | cis |
| CXCL1 | rs3117604 | T | C | -0.28 | 0.01 | 5.80E-117 | 0.01 | 0.02 | 0.48 | 544.44 | cis |
| CXCL6 | rs138723628 | A | G | 0.67 | 0.07 | 7.80E-21 | -0.09 | 0.07 | 0.16 | 86.59 | cis |
| CXCL6 | rs16850073 | T | C | 0.53 | 0.01 | 1.00E-200 | -0.01 | 0.02 | 0.72 | 1950.69 | cis |
| Dkk-1 | rs7898709 | T | G | -0.23 | 0.02 | 1.90E-37 | -0.03 | 0.03 | 0.27 | 163.27 | cis |
| ECP | rs147307766 | T | C | 0.50 | 0.05 | 9.00E-26 | -0.02 | 0.05 | 0.70 | 108.51 | cis |
| EGF | rs2237045 | A | G | -0.13 | 0.02 | 2.00E-13 | 0.01 | 0.02 | 0.68 | 52.16 | cis |
| EN-RAGE | rs11205280 | A | C | 0.17 | 0.02 | 2.70E-17 | -0.03 | 0.03 | 0.37 | 72.25 | cis |
| EN-RAGE | rs3014874 | A | G | -0.17 | 0.02 | 1.60E-29 | -0.02 | 0.02 | 0.47 | 128.44 | cis |
| ESM-1 | rs4865884 | A | G | 0.13 | 0.02 | 2.80E-11 | 0.06 | 0.02 | 0.01 | 42.25 | cis |
| ESM-1 | rs4865911 | A | G | 0.11 | 0.02 | 2.30E-09 | -0.02 | 0.02 | 0.25 | 37.35 | cis |
| FABP4 | rs77878271 | A | G | 0.26 | 0.04 | 6.40E-14 | 0.01 | 0.04 | 0.75 | 55.18 | cis |
| FAS | rs4934436 | T | C | 0.12 | 0.01 | 5.60E-37 | -0.03 | 0.02 | 0.11 | 153.04 | cis |
| FAS | rs982764 | T | C | 0.25 | 0.01 | 1.50E-132 | -0.02 | 0.02 | 0.21 | 625.00 | cis |
| FS | rs31226 | T | C | 0.13 | 0.01 | 1.60E-33 | 0.02 | 0.02 | 0.32 | 139.67 | cis |
| Gal-3 | rs3825615 | T | C | 0.22 | 0.03 | 1.10E-16 | 0.11 | 0.05 | 0.03 | 71.60 | cis |
| Gal-3 | rs76480089 | A | G | 0.60 | 0.02 | 1.00E-200 | -0.02 | 0.03 | 0.63 | 1600.00 | cis |
| GDF-15 | rs1227734 | T | C | 0.37 | 0.01 | 9.90E-177 | 0.02 | 0.03 | 0.52 | 810.06 | cis |
| HB-EGF | rs182055959 | T | C | 0.96 | 0.13 | 1.10E-13 | 0.15 | 0.34 | 0.67 | 54.53 | cis |
| HGF | rs5745687 | T | C | -0.20 | 0.02 | 3.10E-18 | 0.07 | 0.04 | 0.11 | 75.61 | cis |
| hK11 | rs117268623 | T | C | -1.40 | 0.04 | 1.00E-200 | 0.02 | 0.06 | 0.78 | 1012.40 | cis |
| hK11 | rs2691217 | T | C | 0.11 | 0.02 | 4.20E-11 | 0.00 | 0.02 | 0.84 | 41.87 | cis |
| IL-18 | rs5744249 | A | C | 0.22 | 0.01 | 3.70E-74 | -0.02 | 0.02 | 0.43 | 336.11 | cis |
| IL-1ra | rs6734238 | A | G | 0.20 | 0.01 | 2.50E-85 | -0.02 | 0.02 | 0.39 | 400.00 | cis |
| IL-8 | rs7655660 | A | G | -0.19 | 0.02 | 2.70E-15 | 0.08 | 0.05 | 0.09 | 62.67 | cis |
| IL16 | rs4778639 | T | G | 0.96 | 0.02 | 1.00E-200 | -0.02 | 0.04 | 0.57 | 2844.44 | cis |
| KIM-1 | rs6555820 | A | C | -0.48 | 0.01 | 1.00E-200 | 0.00 | 0.02 | 0.99 | 2552.91 | cis |
| KLK6 | rs268891 | A | C | 0.19 | 0.01 | 2.20E-72 | -0.03 | 0.02 | 0.09 | 361.00 | cis |
| LOX-1 | rs10505752 | A | G | 0.11 | 0.02 | 2.40E-12 | 0.07 | 0.02 | 0.01 | 53.78 | cis |
| MMP-10 | rs17860955 | T | C | 1.10 | 0.04 | 6.40E-143 | 0.14 | 0.13 | 0.29 | 625.00 | cis |
| MMP-12 | rs72981675 | T | C | -0.77 | 0.01 | 1.00E-200 | -0.02 | 0.02 | 0.30 | 3025.00 | cis |
| MMP-1 | rs471994 | A | G | -0.36 | 0.01 | 1.00E-200 | 0.04 | 0.02 | 0.06 | 1071.07 | cis |
| MMP-3 | rs4614414 | T | C | -0.09 | 0.01 | 2.30E-10 | 0.03 | 0.03 | 0.24 | 37.73 | cis |
| MMP-3 | rs632478 | T | G | -0.48 | 0.01 | 1.00E-200 | -0.01 | 0.02 | 0.54 | 2908.72 | cis |
| MMP-7 | rs11568819 | A | G | 0.56 | 0.02 | 9.50E-116 | -0.06 | 0.04 | 0.09 | 544.44 | cis |
| MMP-7 | rs11607749 | T | C | -0.08 | 0.01 | 4.20E-10 | 0.02 | 0.02 | 0.22 | 41.17 | cis |
| MPO | rs34097845 | T | C | -0.35 | 0.02 | 2.70E-73 | 0.00 | 0.03 | 0.88 | 339.34 | cis |
| NT-pro_BNP | rs198389 | A | G | -0.21 | 0.02 | 4.40E-41 | 0.02 | 0.02 | 0.23 | 172.27 | cis |
| OPG | rs11300005 | CT | C | -0.20 | 0.01 | 2.50E-79 | -0.04 | 0.02 | 0.03 | 400.00 | cis |
| PAPPA | rs17372936 | T | C | 0.09 | 0.01 | 2.40E-12 | 0.01 | 0.02 | 0.82 | 52.28 | cis |
| PAR-1 | rs250727 | T | C | 0.12 | 0.01 | 1.20E-29 | -0.02 | 0.02 | 0.25 | 119.01 | cis |
| PlGF | rs175510 | A | G | 0.11 | 0.01 | 1.30E-29 | 0.00 | 0.02 | 0.91 | 121.00 | cis |
| PTX3 | rs9859018 | A | G | 0.10 | 0.01 | 4.00E-14 | -0.05 | 0.02 | 0.01 | 51.02 | cis |
| RAGE | rs204993 | A | G | 0.15 | 0.01 | 1.20E-35 | -0.02 | 0.02 | 0.31 | 156.25 | cis |
| REN | rs193280350 | A | G | 0.87 | 0.06 | 1.40E-43 | 0.02 | 0.06 | 0.67 | 190.70 | cis |
| REN | rs78926127 | A | G | 0.38 | 0.05 | 1.10E-13 | 0.00 | 0.05 | 0.94 | 55.52 | cis |
| RETN | rs10401670 | T | C | 0.15 | 0.01 | 9.00E-37 | -0.02 | 0.02 | 0.20 | 156.25 | cis |
| RETN | rs34861192 | A | G | 1.10 | 0.05 | 7.80E-89 | 0.11 | 0.16 | 0.48 | 414.95 | cis |
| SCF | rs11608458 | T | C | -0.07 | 0.01 | 5.90E-09 | -0.01 | 0.02 | 0.60 | 34.03 | cis |
| SELE | rs4656716 | A | G | 0.11 | 0.01 | 6.90E-25 | -0.02 | 0.02 | 0.30 | 121.00 | cis |
| SIRT2 | rs144373891 | T | C | -0.58 | 0.06 | 2.90E-23 | -0.04 | 0.09 | 0.65 | 96.64 | cis |
| TF | rs11589759 | T | C | -0.18 | 0.01 | 6.90E-62 | -0.03 | 0.02 | 0.08 | 267.77 | cis |
| TIE2 | rs35030851 | T | G | 0.82 | 0.03 | 1.00E-200 | -0.12 | 0.07 | 0.07 | 994.67 | cis |
| TM | rs1042579 | A | G | 0.40 | 0.01 | 1.00E-200 | -0.01 | 0.02 | 0.59 | 946.75 | cis |
| TNF-R1 | rs4149584 | T | C | -0.35 | 0.04 | 7.90E-20 | -0.04 | 0.08 | 0.62 | 84.83 | cis |
| TNF-R2 | rs5746026 | A | G | -0.38 | 0.03 | 1.10E-40 | 0.15 | 0.06 | 0.02 | 184.18 | cis |
| TNFSF14 | rs344560 | T | C | -0.63 | 0.03 | 1.60E-137 | 0.03 | 0.04 | 0.38 | 635.04 | cis |
| TNFSF14 | rs413141 | A | G | 0.16 | 0.02 | 8.40E-23 | -0.02 | 0.02 | 0.34 | 100.00 | cis |
| TRANCE | rs4512994 | A | C | 0.12 | 0.01 | 4.60E-29 | 0.00 | 0.02 | 0.93 | 119.01 | cis |
| U-PAR | rs2302524 | T | C | 0.14 | 0.01 | 6.00E-28 | 0.02 | 0.03 | 0.57 | 115.98 | cis |
| U-PAR | rs8104447 | T | C | -0.06 | 0.01 | 5.70E-11 | 0.00 | 0.02 | 0.86 | 43.53 | cis |
| VEGF-A | rs6921438 | A | G | -0.45 | 0.01 | 1.00E-200 | -0.04 | 0.02 | 0.02 | 1406.25 | cis |
| CD40-L | rs150239481 | A | G | 0.99 | 0.17 | 1.60E-08 | 0.37 | 0.68 | 0.59 | 33.91 | pan |
| CD40-L | rs4602861 | A | G | 0.08 | 0.01 | 1.40E-08 | 0.00 | 0.02 | 0.89 | 29.47 | pan |
| CD40 | rs4801216 | T | C | -0.09 | 0.01 | 1.80E-12 | 0.01 | 0.02 | 0.78 | 47.93 | pan |
| CD40 | rs4810485 | T | G | -0.50 | 0.01 | 1.00E-200 | -0.02 | 0.02 | 0.44 | 1736.11 | pan |
| GAL | rs307575 | A | G | -0.12 | 0.02 | 6.20E-13 | 0.01 | 0.02 | 0.77 | 49.83 | pan |
| GAL | rs4672375 | A | G | -0.18 | 0.02 | 3.70E-36 | -0.03 | 0.02 | 0.13 | 144.00 | pan |
| GAL | rs77542162 | A | G | -0.52 | 0.07 | 2.80E-13 | 0.05 | 0.11 | 0.67 | 53.64 | pan |
| GAL | rs9405503 | A | G | -0.17 | 0.03 | 1.70E-10 | 0.05 | 0.03 | 0.12 | 42.75 | pan |
| IL-27 | rs10774624 | A | G | -0.08 | 0.01 | 2.20E-13 | 0.06 | 0.02 | 0.00 | 57.76 | pan |
| IL-27 | rs10843390 | T | C | 0.06 | 0.01 | 3.10E-08 | -0.01 | 0.02 | 0.62 | 31.77 | pan |
| IL-27 | rs11599750 | T | C | -0.18 | 0.01 | 8.00E-66 | 0.04 | 0.02 | 0.04 | 324.00 | pan |
| IL-27 | rs11711157 | T | C | -0.09 | 0.01 | 7.60E-11 | -0.02 | 0.02 | 0.43 | 41.33 | pan |
| IL-27 | rs1257169 | A | C | -0.10 | 0.01 | 1.30E-17 | -0.02 | 0.02 | 0.25 | 77.76 | pan |
| IL-27 | rs12625762 | A | G | 0.08 | 0.02 | 3.20E-08 | 0.00 | 0.03 | 0.87 | 29.88 | pan |
| IL-27 | rs4905 | A | G | 0.67 | 0.01 | 1.00E-200 | 0.02 | 0.02 | 0.29 | 4489.00 | pan |
| IL-27 | rs704 | A | G | -0.13 | 0.01 | 1.40E-39 | -0.02 | 0.02 | 0.17 | 169.00 | pan |
| IL-27 | rs9715769 | A | C | -0.10 | 0.02 | 4.50E-11 | 0.03 | 0.03 | 0.31 | 40.96 | pan |
| IL-6RA | rs12126142 | A | G | 0.96 | 0.01 | 1.00E-200 | -0.03 | 0.02 | 0.15 | 19357.28 | pan |
| IL-6 | rs2228145 | A | C | -0.17 | 0.01 | 3.30E-45 | 0.03 | 0.02 | 0.16 | 200.69 | pan |
| IL-6 | rs4959106 | T | C | -0.08 | 0.01 | 2.40E-09 | 0.00 | 0.02 | 0.95 | 34.31 | pan |
| ITGB1BP2 | rs342287 | T | C | 0.15 | 0.02 | 1.20E-10 | 0.00 | 0.02 | 0.89 | 42.53 | pan |
| MB | rs10741929 | A | C | -0.08 | 0.01 | 2.80E-13 | 0.02 | 0.02 | 0.29 | 50.28 | pan |
| MB | rs375034445 | A | AT | -0.11 | 0.02 | 2.80E-09 | -0.01 | 0.02 | 0.79 | 33.52 | pan |
| PECAM-1 | rs11600151 | T | C | 0.14 | 0.02 | 1.80E-18 | 0.02 | 0.03 | 0.49 | 76.56 | pan |
| SPON1 | rs10832172 | T | C | -0.23 | 0.01 | 6.20E-94 | -0.03 | 0.02 | 0.10 | 437.19 | pan |
| t-PA | rs10744481 | A | G | -0.07 | 0.01 | 8.40E-11 | 0.00 | 0.02 | 0.95 | 40.50 | pan |
| t-PA | rs10965545 | A | G | 0.14 | 0.03 | 1.90E-08 | 0.03 | 0.04 | 0.45 | 28.99 | pan |
| TRAIL | rs2304456 | T | G | 0.26 | 0.02 | 5.10E-43 | -0.01 | 0.03 | 0.60 | 187.26 | pan |
| TRAIL | rs28929474 | T | C | 0.66 | 0.04 | 1.20E-51 | -0.01 | 0.06 | 0.83 | 235.59 | pan |
| TRAIL | rs35617250 | T | C | 0.11 | 0.02 | 2.60E-14 | -0.02 | 0.03 | 0.53 | 53.78 | pan |
| TRAIL | rs4760 | A | G | 0.36 | 0.02 | 1.70E-101 | -0.05 | 0.02 | 0.04 | 448.44 | pan |
| TRAIL | rs673408 | A | G | 0.09 | 0.01 | 1.50E-13 | -0.01 | 0.02 | 0.53 | 51.36 | pan |
| TRAIL | rs8178824 | T | C | 0.26 | 0.04 | 5.80E-14 | 0.07 | 0.09 | 0.45 | 55.18 | pan |
| VEGF-D | rs2731673 | T | C | -0.08 | 0.01 | 2.00E-12 | 0.02 | 0.02 | 0.34 | 45.56 | pan |
| VEGF-D | rs3733402 | A | G | 0.19 | 0.01 | 3.40E-65 | -0.05 | 0.02 | 0.01 | 298.35 | pan |
| VEGF-D | rs5030073 | A | G | -0.10 | 0.01 | 1.40E-19 | -0.04 | 0.02 | 0.02 | 81.00 | pan |
| VEGF-D | rs7178801 | A | G | 0.06 | 0.01 | 4.40E-08 | -0.03 | 0.02 | 0.08 | 27.56 | pan |

**Table S2** Heterogeneity and pleiotropy test of MMP-10

| Heterogeneity test |  |  |  |  |
| --- | --- | --- | --- | --- |
| exposure | method | Q | Q_df | Q_pval |
| MMP-10 | MR Egger | 0.008 | 1 | 0.929 |
| MMP-10 | Inverse variance weighted | 0.478 | 2 | 0.787 |
| Pleiotropy test |  |  |  |  |
| exposure | method | egger_intercept | se | pval |
| MMP-10 | MR Egger | 0.013 | 0.020 | 0.617 |

**Table S3** All IVs for drug-target mendelian randomization

|  |  |  |  | exposure | | | | outcome | | |  |  |
| --- | --- | --- | --- | --- | --- | --- | --- | --- | --- | --- | --- | --- |
| protein | SNP | effect allele | other allele | beta | se | p | beta | | se | p | F value | type of IVs |
| VEGF R1 | rs56728557 | A | G | -0.37 | 0.02 | 7.84E-113 | 0.08 | | 0.02 | 0.00 | 647.32 | lead cis-eQTL |
| VEGF sR2 | rs34231037 | G | A | -1.15 | 0.06 | 1.00E-70 | -0.04 | | 0.05 | 0.40 | 310.34 | cis-pQTL |
| VEGF sR3 | rs34221241 | C | T | -0.36 | 0.04 | 1.50E-19 | -0.01 | | 0.03 | 0.84 | 85.52 | cis-pQTL |
| VEGF sR2 | rs635634 | T | C | -0.58 | 0.03 | 4.0E-80 | -0.01 | | 0.02 | 0.78 | 355.96 | trans-pQTL |
| VEGF sR3 | rs10935473 | T | G | -0.69 | 0.02 | 1.0E-200 | -0.03 | | 0.02 | 0.16 | 485.62 | trans-pQTL |

**Table S4** Statistical results of differential expression of 8 target protein genes between cancer and healthy controls

| gene | group | n | min | max | median | iqr | q1 | q3 | mean | sd | se |
| --- | --- | --- | --- | --- | --- | --- | --- | --- | --- | --- | --- |
| OLR1 | Normal | 41.00 | 0.11 | 1.09 | 0.47 | 0.37 | 0.29 | 0.66 | 0.50 | 0.26 | 0.04 |
| OLR1 | Tumor | 480.00 | 0.00 | 7.76 | 1.96 | 2.06 | 1.14 | 3.19 | 2.25 | 1.43 | 0.07 |
| VEGFA | Normal | 41.00 | 2.64 | 4.70 | 3.61 | 0.63 | 3.21 | 3.84 | 3.57 | 0.46 | 0.07 |
| VEGFA | Tumor | 480.00 | 2.97 | 7.43 | 5.16 | 0.89 | 4.72 | 5.61 | 5.17 | 0.69 | 0.03 |
| TNFRSF11B | Normal | 41.00 | 1.07 | 3.65 | 2.55 | 0.65 | 2.28 | 2.92 | 2.56 | 0.53 | 0.08 |
| TNFRSF11B | Tumor | 480.00 | 0.36 | 9.19 | 3.74 | 1.66 | 2.97 | 4.63 | 3.85 | 1.45 | 0.07 |
| PTX3 | Normal | 41.00 | 1.07 | 3.60 | 1.78 | 0.87 | 1.54 | 2.41 | 1.96 | 0.65 | 0.10 |
| PTX3 | Tumor | 480.00 | 0.00 | 4.73 | 0.78 | 0.69 | 0.48 | 1.17 | 0.93 | 0.69 | 0.03 |
| TNFRSF1B | Normal | 41.00 | 5.00 | 6.75 | 5.73 | 0.47 | 5.49 | 5.96 | 5.71 | 0.39 | 0.06 |
| TNFRSF1B | Tumor | 480.00 | 2.37 | 7.89 | 6.02 | 1.04 | 5.45 | 6.49 | 5.92 | 0.78 | 0.04 |
| MMP7 | Normal | 41.00 | 0.00 | 3.24 | 0.27 | 0.45 | 0.13 | 0.58 | 0.47 | 0.62 | 0.10 |
| MMP7 | Tumor | 480.00 | 0.15 | 11.16 | 5.39 | 3.09 | 3.76 | 6.85 | 5.29 | 2.17 | 0.10 |
| ADM | Normal | 41.00 | 3.42 | 6.94 | 4.96 | 0.80 | 4.60 | 5.40 | 5.04 | 0.73 | 0.11 |
| ADM | Tumor | 480.00 | 2.12 | 7.73 | 4.86 | 1.43 | 4.11 | 5.55 | 4.85 | 1.01 | 0.05 |
| MMP10 | Normal | 41.00 | 0.00 | 5.07 | 0.37 | 0.47 | 0.25 | 0.72 | 0.63 | 0.85 | 0.13 |
| MMP10 | Tumor | 480.00 | 0.00 | 7.41 | 2.39 | 1.98 | 1.37 | 3.35 | 2.41 | 1.37 | 0.06 |

**Table S5** Statistical results of ROC curves of 6 target protein genes based on clinical information

| gene | outcome | AUC | CI |
| --- | --- | --- | --- |
| OLR1 | forward | 0.917 | 0.891 - 0.943 |
| VEGFA | forward | 0.976 | 0.960 - 0.992 |
| TNFRSF11B | forward | 0.818 | 0.778 - 0.858 |
| PTX3 | reverse | 0.889 | 0.858 - 0.920 |
| TNFRSF1B | forward | 0.630 | 0.567 - 0.692 |
| MMP7 | forward | 0.984 | 0.972 - 0.995 |
| MMP10 | forward | 0.885 | 0.831 - 0.939 |

**Figure S1** Mendelian randomization analysis results of ADM and MMP-10


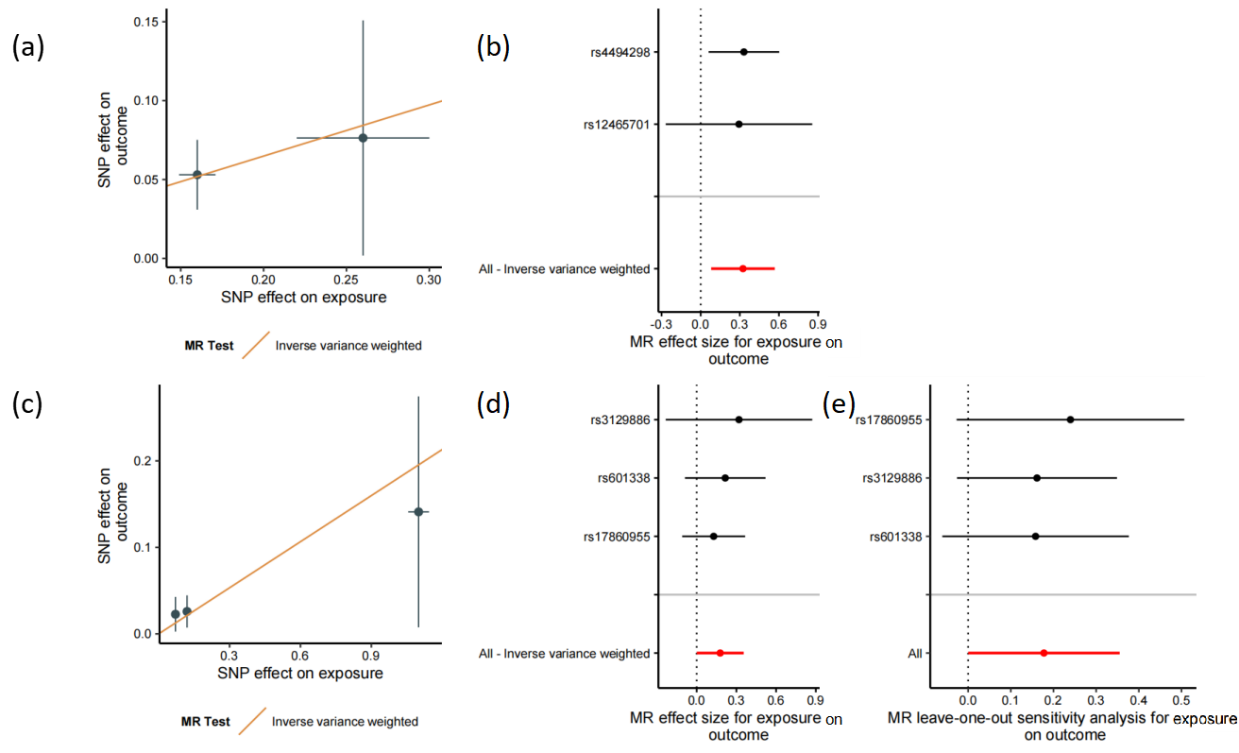


Scatter plot of SNP effects for genetically predicted protein levels and risk of CRC for ADM (a), MMP-10 (c); and forest plot of MR effect for exposure on the outcome ADM (b), MMP-10 (d); and leave-one-out analysis result for MMP-10 (e).
